# Safety Risk Analysis of a New Design of Basalt Fiber Gabion Slope Based on Improved 3D Discrete Element Method and Monitoring Data

**DOI:** 10.3390/s22103645

**Published:** 2022-05-10

**Authors:** Jianjian Dai, Xiangyang Xu, Hao Yang, Chao Su, Nan Ye

**Affiliations:** 1 School of Rail Transit, Soochow University, Suzhou 215006, China; dudud8611@gmail.com; 2 School of Transportation and Civil Engineering, Nantong University, Nantong 226019, China; h.yang@ntu.edu.cn; 3 College of Water Conservancy and Hydropower Engineering, Hohai University, Nanjing 210098, China; csu_hhu@126.com; 4 Jilin Institute of Water Resources Research, Changchun 130022, China; naney_ken@163.com

**Keywords:** gabion, basalt fiber reinforcement, DEM, seepage, deformation analysis, stability

## Abstract

Gabion has been extensively used in retaining walls and slope protection. This study carries out a safety risk analysis of a new structure combining basalt fiber reinforcement (BFR) and the traditional gabion structure. The micro-parameters of BFR and soil were calibrated by using the 3D discrete element method after the tensile test of BFR was completed. The mechanical property of the gabion unit was investigated by using a refined model and a numerical test of uniaxial compression. This work developed a simplified method to simulate the seepage effect. The stress condition and sliding displacement between gabions were also investigated. Deformation, stress, and porosity were all used to evaluate the stability of the new type of gabion slope. According to this study, BFR has a tensile strength of 68.22 MPa, and the safety factor increased by 25.68% after using these BFR gabions. The damage is mainly manifested by bending the BFRs and the dislocation of the gabion units, as the slope does not slip. It is indicated this novel gabion structure has a lower safety risk compared to traditional ones, and thus can be popularized and used in retaining walls and slope protection.

## 1. Introduction

Gabions have the advantages of strong adaptability, convenient construction, and low cost. They are widely used in geotechnical designs, such as impact load buffering [1,2,3,4], retaining walls [5], river slope protection [6,7], and overflow weir [8,9,10,11]. Many researchers have carried out tests to explore the mechanical properties of the gabion. For example, Lambert et al. [12] and Perera et al. [13] carried out on-site impact tests to study the mechanical response of gabion as a buffer layer. Ng et al. [14,15] carried out an impact energy of 70 kJ field test on a gabion retaining wall with a thickness of 1 m to study the response of the stone cage under six consecutive impacts. The results showed that after six successive hits at an energy level of 70 kJ, the force transmitted increased by 40%. Gabion retaining walls rely primarily on rock debris rearrangement to absorb impact energy. This research is helpful for practitioners when designing rigid barriers. The gabion’s field impact test model has a large scale and a short load time. It is excellent to use monitoring technology based on the Internet of things [16], which is convenient for data monitoring and improving accuracy and is worth further exploration. The mechanical characteristics of the gabion were investigated by Jiang Y et al. [17] under uniaxial compression and direct shear conditions (the sample size was 0.5 m × 0.5 m × 0.4 m). Samayoa et al. [18] used theoretical analysis and numerical simulation to study the seismic performance of gabion retaining walls. Nishold et al. [19] used a physical model to assess the use of a geo-tube with gabions in coastal protection and then used it to defend the shore. Cho et al. [20] conducted model tests on the slope, examining the damage caused by heavy rain by placing gabions and drainage materials at the slope’s toe. This study found that preventing the diving surface on the slope surface is critical. The slope’s stability can be improved by strategically placing gabions and drainage pipes. Moss et al. [21] pointed out that in cavern engineering when the rock mass deformed after installing the brace, the displacement capacity of the prop was being consumed. It is necessary to design the support capacity remaining when support is required. The research showed that using a “gabion type” support system could achieve these goals and introduced the technical process of “gabion panel” support system design. This research provided a new rock support design method. Currently, gabion research mainly focuses on the traditional gabion structure, and there is no study on new or enhanced gabion structures.

As a typical scattered body, the boulders filled in the gabion had significant discontinuous properties. The discrete element method (DEM) [22] is an excellent tool for modeling gabion structures because it is a discontinuous medium. Bertrand et al. [23,24] calibrated the micro-parameters of the double-twisted hexagonal mesh by comparing simulations to experimental tensile strength tests based on DEM. The impact load effect of rockfall protection was simulated, and the results were analyzed from an engineering point of view. Su et al. [25] used DEM to study the effect of particle size and buffer thickness on impact load and transmitted load. The smaller particle size in the buffer layer, the force chain collapses easier, and the buffer layer thickness should be three times the radius of the pebble to remove the effect of energy reflecting from the barrier back to the point of impact. Su et al. [26] employed DEM to simulate gabions’ impact resistance and cushioning capability with four different sphericity particle shapes. The study showed that the penetration depth of the pebble decreased with the increase of the particle angle, and the load diffusion angle of the circular particle was 20°. Furthermore, Su et al. [27] studied the combined effect of rock shape (bulky and flaky) and compressive strength (20 MPa to 50 MPa) on the dynamic response of the gabion when the pebble’s impact energy was 70 kJ. The study showed that rounder rocks should be used to reduce the concentrated impact load, and the maximum impact and transfer load of flaky rocks is about 20% larger than that of bulky rocks. In general, the DEM is suitable for studying the gabion structures.

Compared with the iron wire of traditional gabion structures, basalt fiber reinforcement (BFR) is a continuous fiber material obtained from natural basalt, which has outstanding features of high strength and corrosion resistance [28] and can be degraded in the environment after being discarded. In this study, the BFR is integrated into the gabion structure. First, a tensile test determined the tensile strength of BFR, and the micro-parameters of gabion and soil were calibrated. The uniaxial compression characteristics of gabion were simulated by the commercial discrete element software PFC3D. Then the simplified simulation method of the seepage effect was studied, and a 3D DEM model of the BFR gabion slope was established. Finally, the gabion structures, as well as the stability of the slope, were investigated.

## 2. Tensile Test and Calibration

### 2.1. Tensile Test of BFR

**Figure 1:**
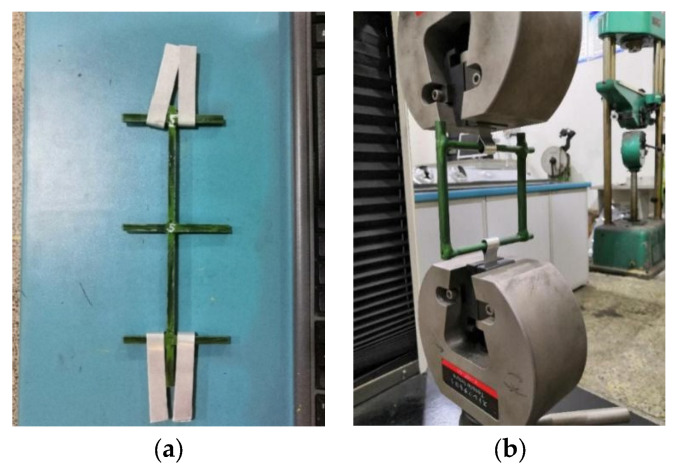
Sample of tensile test. (**a**) Single BFR; (**b**) BFR mesh.

This tensile test is to obtain the tensile strength of a single BFR and the maximum tensile force of the BFR mesh. In this test, the single BFR is 110 mm to 115 mm in length with a diameter of 4 mm to 6 mm. Due to the limitation of the chuck of the test system, the anchoring depth is no more than 50 mm. The fixing method for retaining the cross node is shown in Figure 1a. The mesh length of BFR is 0.05 m. Tensioning is achieved by fixing two aluminum sheets in the middle of the top and bottom of the mesh, with a 2 mm/min tensile speed during the test.

**Figure 2:**
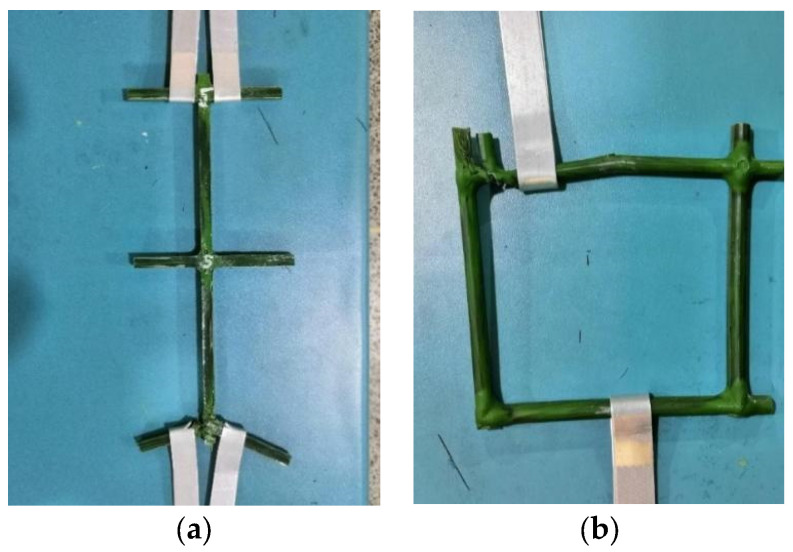
Failure of specimen. (**a**) Single BFR. (**b**) BFR mesh.

The sample is shown in Figure 1b. This test shows that the tensile strength of a single BFR is 86 MPa, and the results are detailed in Table 1. The maximum tensile force of BFR mesh is 1339.5 N (the corresponding tensile strength is 68.22 MPa). Figure 2 shows the failure state after this test.

### 2.2. Micro-Parameters Calibration

#### 2.2.1. BFR

**Figure 3:**
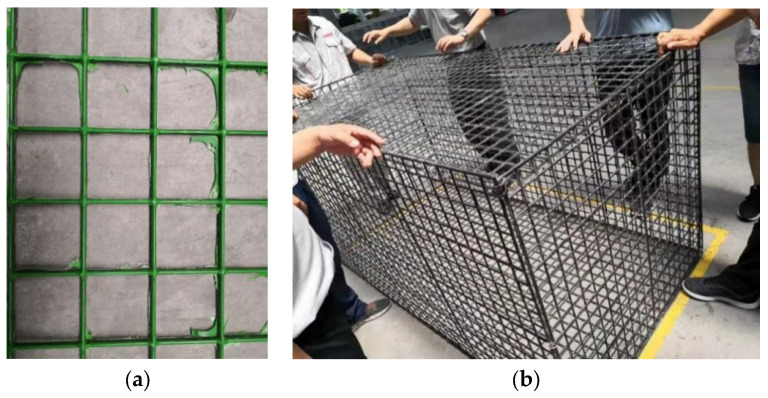
BFR Gabion cage. (**a**) The local model. (**b**) The whole model.

As shown in Figure 3, the gabion cage is made of BFR with a 4 mm to 6 mm diameter. Because the clamping end is easily broken when extended, the BFR mesh results are used for calibration. The mechanical parameters of the gabion and soil [29] are shown in Table 2. The BFR adopts a parallel bonding model, which can convey force and bending moment while simulating tensile and bending properties [30]. The micro-parameters to be calibrated are normal and shear stiffness, normal and shear stiffness of parallel bonding, tensile strength, and cohesion.

**Figure 4:**
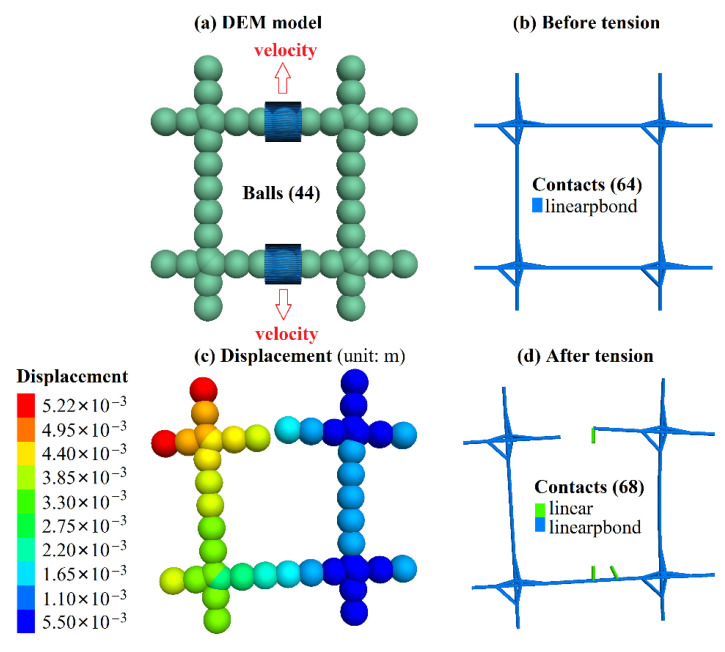
BFR mesh simulated by PFC3D.

The calibration procedure needs to provide a set of micro-parameters by using PFC3D for simulating the tensile test, and continuously adjusting the micro-parameters until the tensile strength obtained by PFC3D is close to the test value. The discrete element model of the tensile test of BFR mesh is shown in Figure 4a. This model is made up of 44 balls loaded by applying velocity to the wall in the PFC3D.

Table 3 shows the final calibrated micro-parameters after lots of trial simulations. The calibrated tensile strength is 66.11 MPa, close to the test value of 68.22 MPa. Figure 4b,c shows the change of contact state, displacement, and failure of the BFR mesh. During the tensile process, the displacement on both sides of the BFR mesh develops on both sides, and this sample breaks from the middle of the BFR mesh’s top, whereas the bottom BFR is not damaged, as shown in Figure 4c. In the tensile test of the BFR mesh, the top-left node location broke, and there were also partial fractures in the middle. The lower part of the sample is intact. The differences between tests and simulations may be due to the fact that the bonding strength of each particle in PFC3D is the same. However, the strength of the joint in the actual manufacturing process cannot be guaranteed due to welding and other reasons.

**Figure 5:**
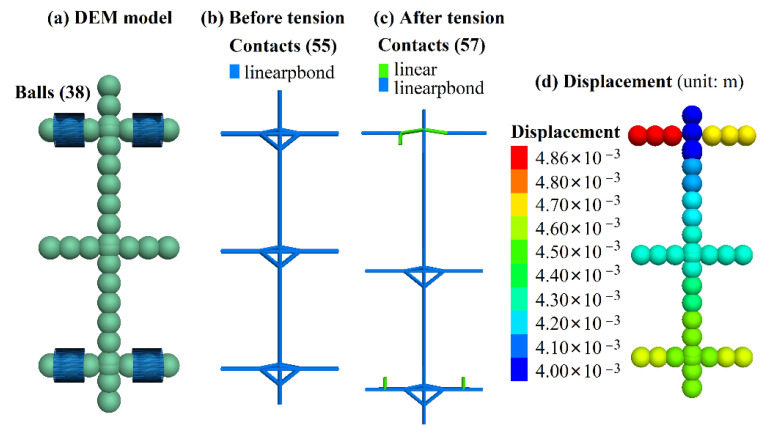
Single BFR simulated by PFC3D.

In addition, we carried out a numerical tensile test of a single BFR with the calibrated micro-parameters above to verify its rationality. The discrete element model of a single BFR with 38 balls is shown in Figure 5a. The tensile strength obtained by the PFC3D is 141.33 MPa. This value is within the range of the test values as shown in Table 1, and the break simulated (Figure 5c) was consistent with that shown in Figure 2.

**Figure 6:**
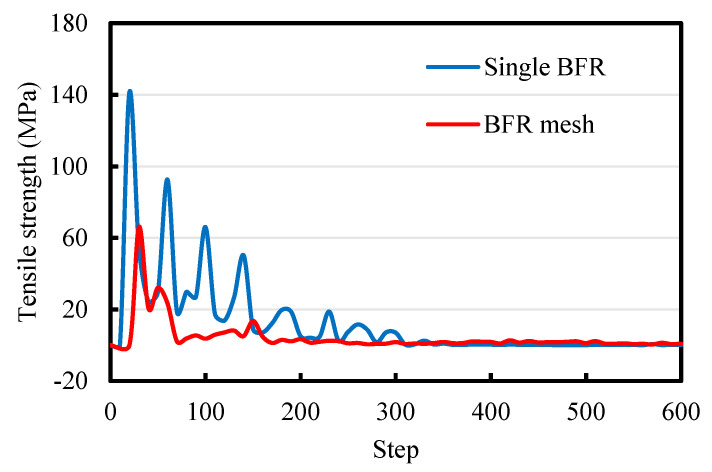
Stress curves simulated by PFC3D.

Figure 6 shows the tensile strength curves of the single BFR and BFR mesh simulated by PFC3D. The abscissa represents the time step. The tensile failure mode of BFR is a brittle fracture, which is supported by the test results.

#### 2.2.2. Soil

**Figure 7:**
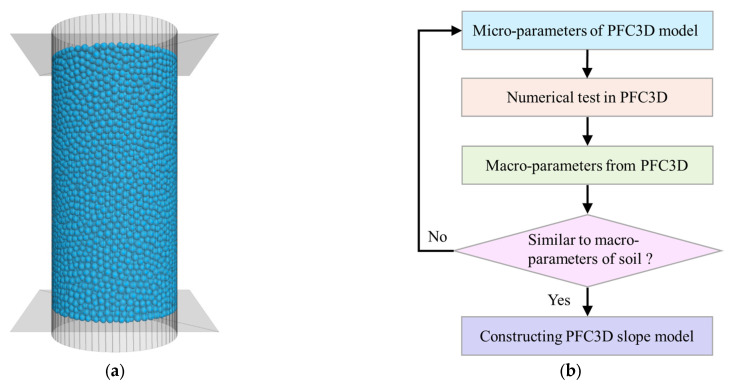

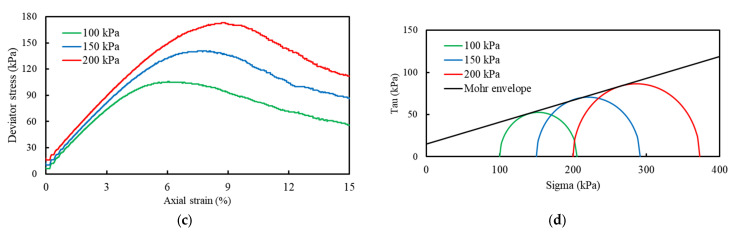
Triaxial compression test of soil in PFC3D. (**a**) Sample. (**b**) The calculation procedure. (**c**) Deviator stress curves. (**d**) Mohr circles and envelope line.

There are no unified formulas between micro- and macro-parameters of cohesive soil. As a result, it is necessary to calibrate the micro-parameters used in PFC3D by numerical triaxial tests. In this study, triaxial compression tests (with confining pressures of 100 kPa, 150 kPa, and 200 kPa) were used to calibrate the micro-parameters of soil. The sample of 16,326 balls is shown in Figure 7a with a size of 2.4 m × 4.8 m (diameter × height). We adopted the contact bond model for soil, which can truly reflect the mechanical properties of clay materials [31,32]. In PFC3D, the idea of obtaining soil’s micro-parameters is as shown in Figure 7b. Table 4 shows the final calibrated micro-parameters.

According to the deviator stress−axial strain curves of 100 kPa, 150 kPa, and 200 kPa confining pressure, the Mohr circles and their envelope are drawn in Figure 7c. The deviator stress-axial strain curve of the soil is also shown in Figure 7d. The shear strength parameters are calculated with the cohesion c = 15.11 kPa and the friction angle φ = 14.57°, which are in good agreement with the values in Table 1 (c = 15.00 kPa and φ = 14.00°).

### 2.3. Uniaxial Compression Simulation of Gabion

**Figure 8:**
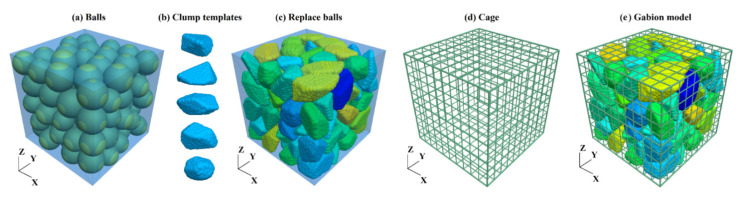
Modeling process of the gabion.

Currently, there are no mechanical tests of gabion with actual size. In this section, the above-calibrated micro-parameters are used to carry out a numerical simulation of the uniaxial compression test of the gabion unit. As shown in Figure 8, the modeling process of the refined gabion is as follows: (1) generate spherical balls of the same size as the rock block. (2) Generate several clump templates to simulate the irregular shape of the rock block (5 templates are selected in this study). (3) Randomly replace the spherical balls with clump templates. (4) Write a function to generate the cage by using the PFC3D built-in programming language FISH; enter the coordinates of gabion’s two diagonal points to generate the cage automatically. (5) Assign micro-parameters to different materials and interfaces.

**Figure 9:**
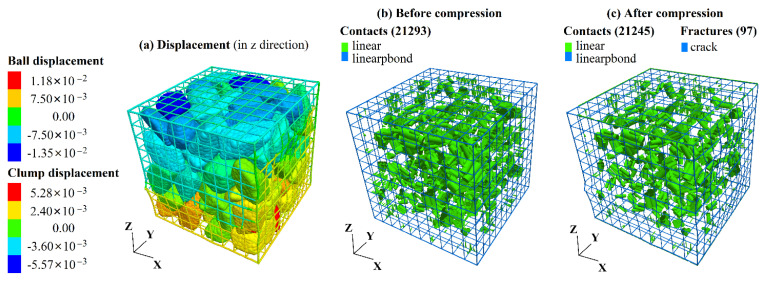
Uniaxial compression simulation in PFC3D.

**Figure 10:**
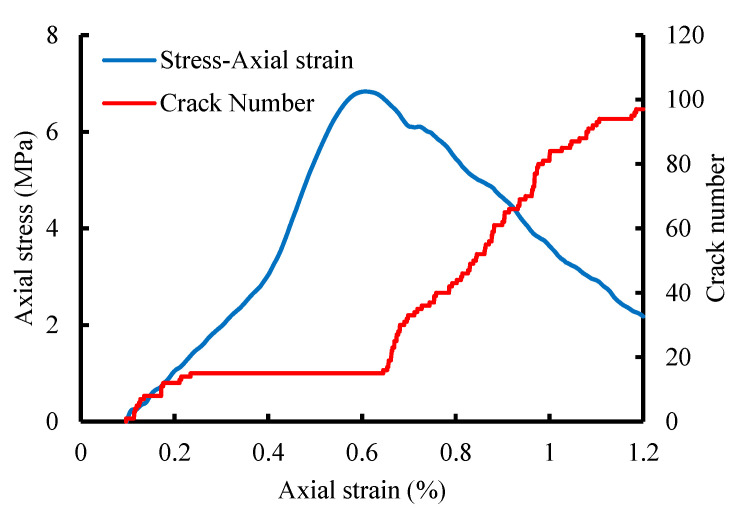
Axial stress-axial strain curve.

In this simulation, the gabion unit’s size is 1 m × 1 m × 1 m (length × width × height), and the loading is realized by applying velocity to the wall. The micro-parameters of the gabion [29] are shown in Table 5. Figure 9 shows the displacement and contact state. The failure mode of the gabion under uniaxial compression is the brittle fracture of BFR. During the failure process, the position of the stone is dislocated, and the number of contacts changes. The number of contacts is 21,293 before compression and reduces to 21,245 after compression. Figure 10 shows the stress-strain curve and the number of cracks during uniaxial compression.

We can see from Figure 10: (1) when the axial strain is less than 0.65%, the axial stress continues to increase with the loading, and the number of cracks increases from 0 to 15. (2) When the axial strain is between 0.23% and 0.65%, the number of cracks is 15. (3) When the axial strain reaches 0.65%, the peak stress is 6.83 MPa. Then the stress decreases, the damage worsens, and the number of cracks increases linearly. (4) As the axial strain increases from 0.65% to 1.20%, cracks increase from 15 to 97, causing the gabion unit to be destroyed.

## 3. 3D Discrete Element Modeling

### 3.1. Simulation of Seepage

Because there are no commands and functions for solving seepage in PFC3D directly, the DEM-CFD coupling method [33,34], such as considering the built-in CFD module in PFC3D or with an external fluid solver. For regular boundaries, this method may be implemented more quickly, but it is tough to deal with complex geometries. Furthermore, it costs lots of time to calculate, and it is hard to debug.

**Figure 11:**
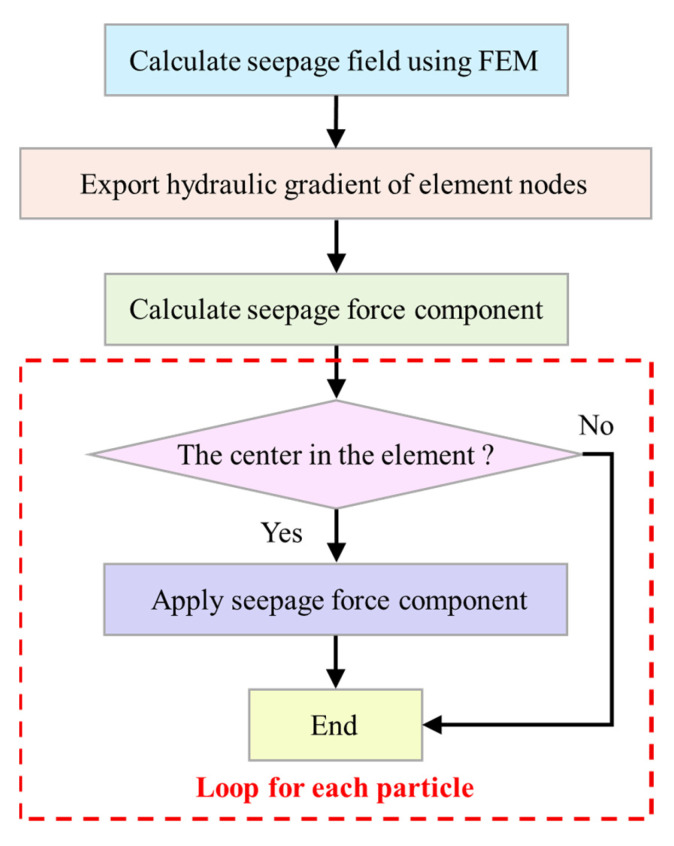
Procedure of seepage force application.

Force is a fundamental solution variable in PFC3D. From the point of view of force, the simplified simulation of seepage can be realized by applying seepage force, which is a kind of body force. Considering the large slope scale, the particle size range in the PFC3D simulation is between centimeter and meter [35,36,37]. In this study, it is assumed that the mesoscopic force of the particles in such a range has little contribution to the whole, so when considering the effect of water, only the rising force and seepage force are considered. For any particle in water, the forces applied to the particle include contact force, frictional force, gravity, rising force, and seepage force. It is only necessary to apply rising and seepage forces to the particle. The FEM is used to calculate the seepage field and export the hydraulic gradient of every node. Then loop for each particle and apply the seepage force. The whole calculating procedure is shown in Figure 11.

**Figure 12:**
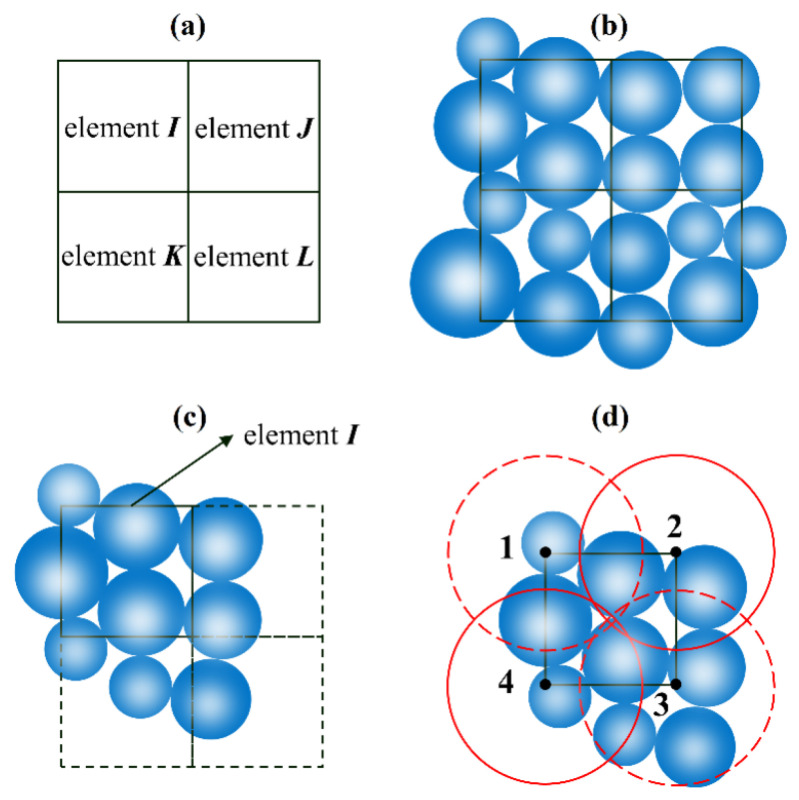
Seepage force of the particle. (**a**) Element number. (**b**) Position of elements and particles. (**c**) Element of loop operation. (**d**) Search range of element.

The method for judging the particle center in the element is shown in Figure 12, taking the four finite elements I, J, K, and L as an example. There will be a specific number of particles spread throughout the element in the same computational domain. For element I, the node is the sphere’s center with a specific radius. The only satisfying requirement is that the sphere at the node can cover the whole element. If the particle’s center is inside the sphere, the component of seepage force is applied to the particle; otherwise not applied. To better simulate the seepage effect, the mesh size of the finite element model should be small and evenly divided. Here are formulas for calculating the seepage force component:

(1) $$F_{x}=\rho_{w}\;g\;i_{x}\;V,$$

(2) $$F_{y}=\rho_{w}\;g\;i_{y}\;V,$$

(3) $$F_{z}=\rho_{w}\;g\;i_{z}\;V.$$

**Figure 13:**
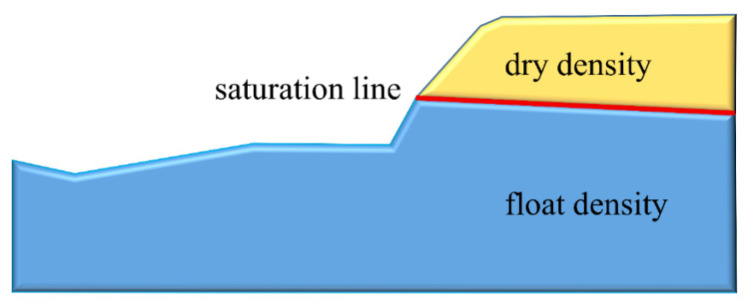
Influence of the rising force.

Furthermore, because the particles below the saturation line calculated by FEM are affected by rising force, the particle adopts the buoyant density as shown in Figure 13.

### 3.2. BFR Gabion Slope

**Figure 14:**
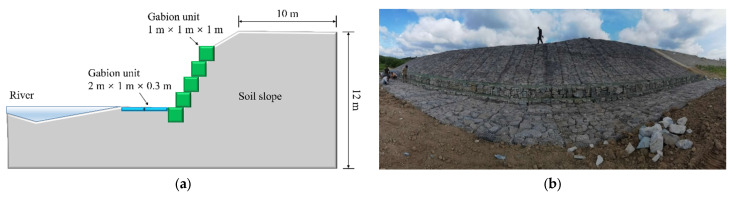
BFR gabion slope. (**a**) Section of the slope. (**b**) Photo of the actual project.

A saline-alkali soil river in Jilin Province, China, has subsidence, seepage, and soil erosion problems. BFR gabions are used for slope protection to obtain local materials, as a means of environmental protection, and to reduce cost. The layout scheme is to place a BFR gabion at the foot of the slope with a size of 1 m × 1 m × 1 m (length × width × height) and two BFR gabions at the left side of the foot of the slope on a size of 2 m × 1 m × 0.3 m. BFR gabions are arranged on the slope surface, and the gabion cages are filled with rock blocks. The schematic diagram of slope protection is shown in Figure 14a.

**Figure 15:**
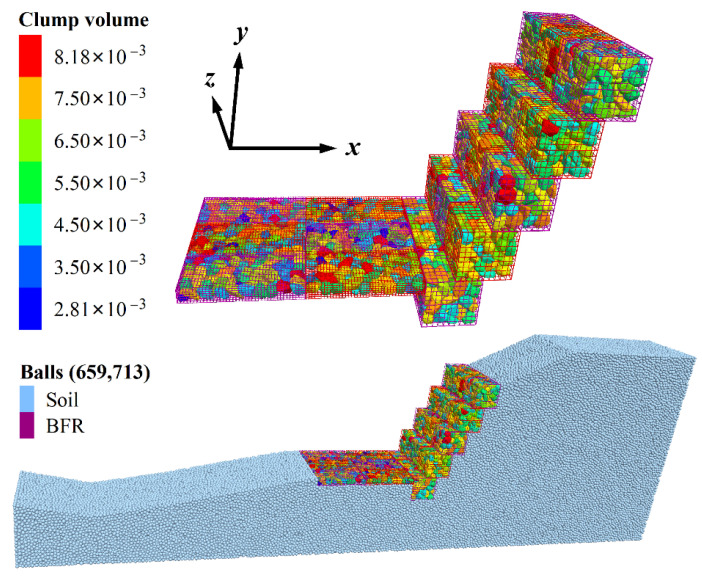
3D DEM model of the BFR gabion slope. The unit of volume is m^3^.

**Figure 16:**
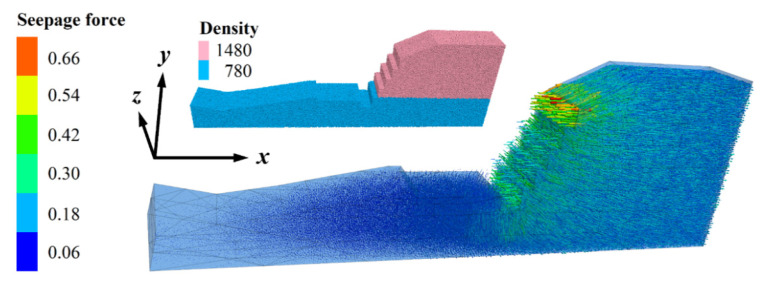
Seepage force and density distribution. The units of seepage force and density are N and kg/m^3^, respectively.

According to the gabion slope’s section, the top of the slope extends 6 m to the right, the thickness direction is 3 m, and the height is 8.84 m. The total number of particles is 659,713, with 276,673 soil particles. The contact between soil and BFRs (C1) or rock blocks (C2) and gabion cages (C3) adopts the linear contact model, and the micro-parameters are shown in Table 6 [29]. The finally established 3D discrete element model is shown in Figure 15. The slope protection part comprises two different sizes of the gabion cage, including fifteen 1 m × 1 m × 1 m (length × width × height) and nine 2 m × 1 m × 0.3 m gabion cages. Block stones distribute randomly, and their volumes range from 2.81 × 10^−3^ m^3^ to 8.18 × 10^−3^ m^3^. The density distribution of soil and seepage force applied is shown in Figure 16. The direction of seepage force is in the slope, and its size ranges from 0.06 to 0.66 N. The density of soil particles above and below the saturation line is 1480 kg/m^3^ and 780 kg/m^3^, respectively.

## 4. Analysis of Gabion Slope

### 4.1. Gabion Structure

#### 4.1.1. Stress State

**Figure 17:**
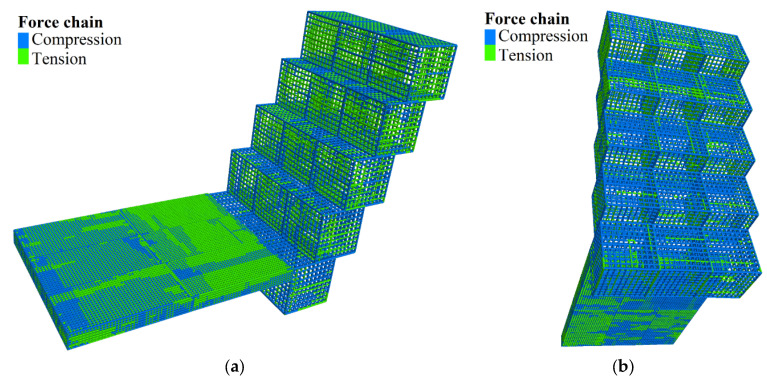
Stress state of the BFR cages. (**a**) Positive side. (**b**) Backside.

**Figure 18:**
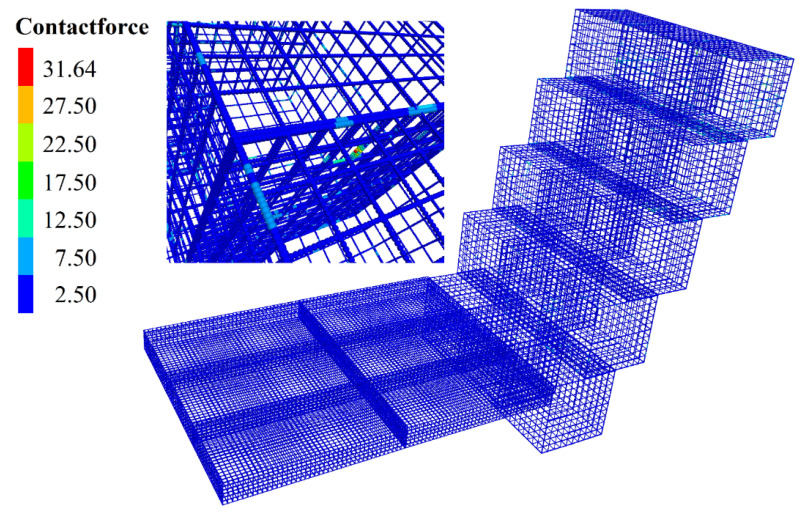
Contact force of the BFRs. The unit of contact force is N.

Figure 17 shows the stress state of gabion cages with green for compression and blue for tension. The BFRs on the gabion’s outer contour and the BFRs on the backside are mainly compressed. The BFRs on the front left and right sides are mainly under tension because of the gravity of the previous gabion. The cages exposed on the outside tend to bulge and deform outwards. The contact force of BFRs is shown in Figure 18. The maximum contact force is 31.64 N, which appears at the second upper part of the cage on the first layer. The tensile stress is 1.61 MPa (divide the contact force by the maximum cross-sectional area of BFR), which is lower than its tensile strength.

#### 4.1.2. Sliding Analysis

**Figure 19:**
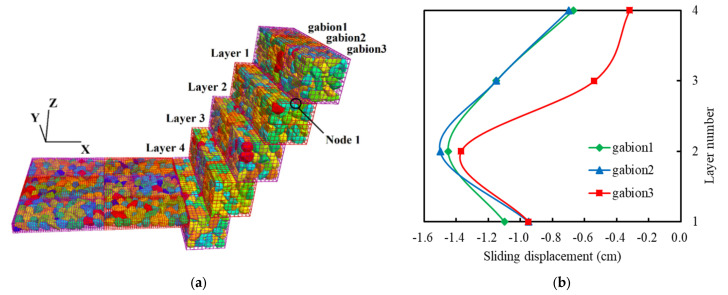
Slip displacement of BFR gabions. (**a**) Schematic diagram. (**b**) Sliding displacement.

In order to describe the contact sliding problem between different layers of BFR gabions more efficiently, we numbered the gabion cages. There are four layers contacting each other, and we choose one vertex at the bottom of the cage named node 1, as shown in Figure 19a.

The slip displacement is calculated by subtracting the x coordinate of node 1 from the x coordinate of the initial model when the calculation is completed. If the relative displacement value is positive, it indicates that the gabion slips in the positive direction of the x-axis; otherwise, the slippage is in the opposite direction. As shown in Figure 19b, the maximum slip is −1.5 cm, located at node 1 of the second gabion on the first floor. The slip displacement curve of three gabions in different layers is consistent. The sliding displacement increases gradually from the first floor to the third floor because the third floor is close to the slope’s toe, the gabion’s overlying load is significant, and the landslide thrust is pretty large. The gabion of the fourth layer is constrained by soil and horizontal gabions, so its slip displacement is smaller than that of the third layer.

### 4.2. Stability Analysis

The basic idea of the strength-reduction method [38] in PFC3D is to reduce the tensile and shear bonding strength and friction coefficient of the particles simultaneously. To analyze the gabion slope better, the safety factor of the original slope obtained by the strength-reduction method is 1.40, and the safety factor of the gabion slope is 1.78. It can be seen that the slope’s safety factor has increased by 25.68% after using BFR gabions.

#### 4.2.1. Deformation Analysis

**Figure 20:**
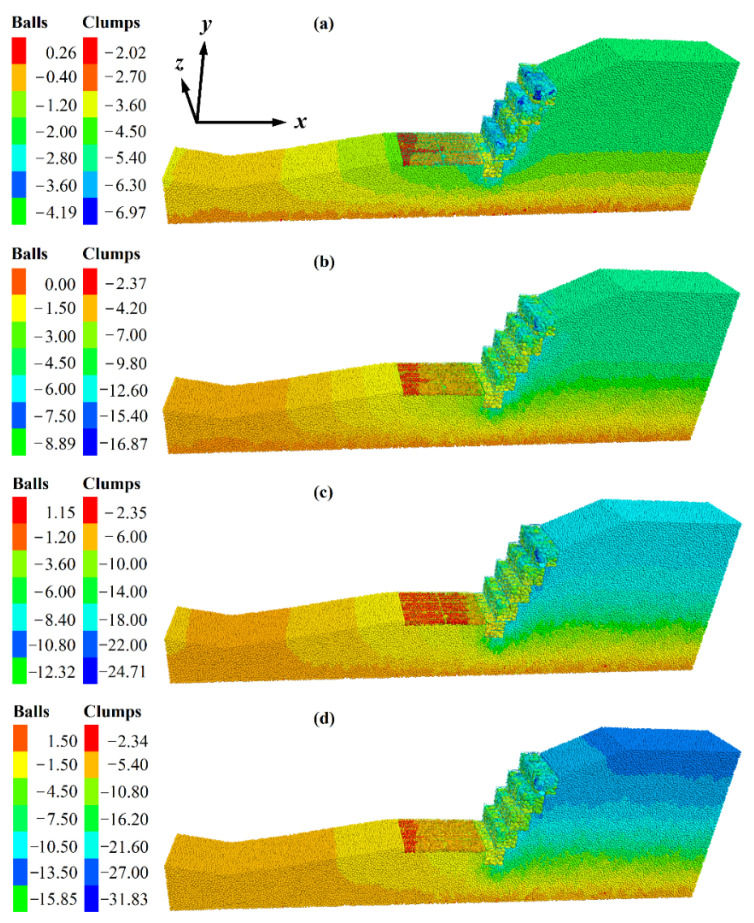
Displacement of the gabion slope. (**a**) 400,000 steps. (**b**) 600,000 steps. (**c**) 800,000 steps. (**d**) 1,000,000 steps. The unit of displacement is cm.

At 400,000 steps, under the influence of gravity and seepage force, the displacement of soil particles gradually developed from the bottom to the top of the slope. The BFR began to occur bending (Figure 20a). At 600,000 steps, the displacement developed toward the middle of the slope. Due to the extrusion of surrounding soil particles, the gabion cage located on the left side of the slope foot began to shift, as shown in Figure 20b. At 800,000 steps, the displacement developed toward the top of the slope. Because of the settlement of the surrounding soil particles, the gabion’s cage was bent, as shown in Figure 20c. At 1 million steps, the displacement was stable, and the displacement at the top of the slope was the largest. The separation of the gabion units appeared (Figure 20d), and no through-sliding surface could have been formed on the slope, and no landslide happened.

#### 4.2.2. Stress Analysis

**Figure 21:**
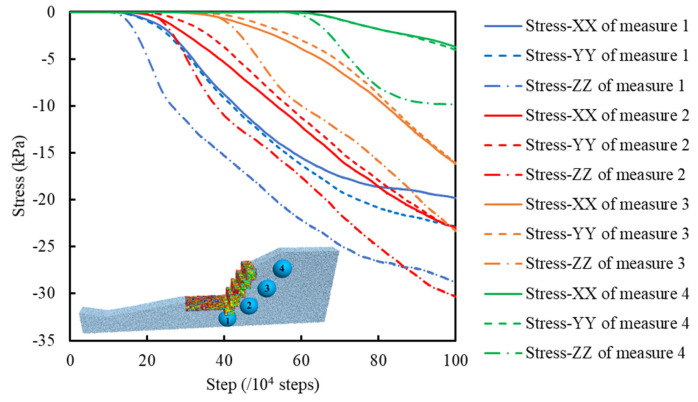
Curve of measured stresses.

To study the change of stress and porosity in the slope, measuring circles with a radius of 1.0 m were set at 4 different positions of the gabion slope, as shown in Figure 21.

Figure 21 shows the recorded stress curves. The stress in the 4 measure circles’ x-, y-, and z-directions were zero at the beginning of the calculation because the displacement of soil particles gradually increased from the toe to the top of the slope. It took time to develop the displacement. The soil particles had reached the equilibrium state before the gabion slope model was established, so the stresses obtained by the measuring circle were near zero at the beginning. No.4 measure circle is close to the top of the slope, and the No.1 measure circle is close to the foot of the slope. As a result, the stresses in the x-, y-, and z-directions of the No.4 measure circle were zero for the longest time, and the No.1 measure circle was the shortest. As the time step increased, under the influence of the gravity of BFR gabions, the soil particles were in close contact and led the recorded stresses in the x-, y-, and z-directions to increase gradually.

We can see that the stress in the z-direction of the measuring circles is more significant than that in the x and y directions. Under the support of BFR gabions, the broken bonds of soil particles could not form a sliding surface. The soil particles were mainly affected by their gravity, seepage force, and the gravity of BFR gabions.

#### 4.2.3. Porosity Analysis

**Figure 22:**
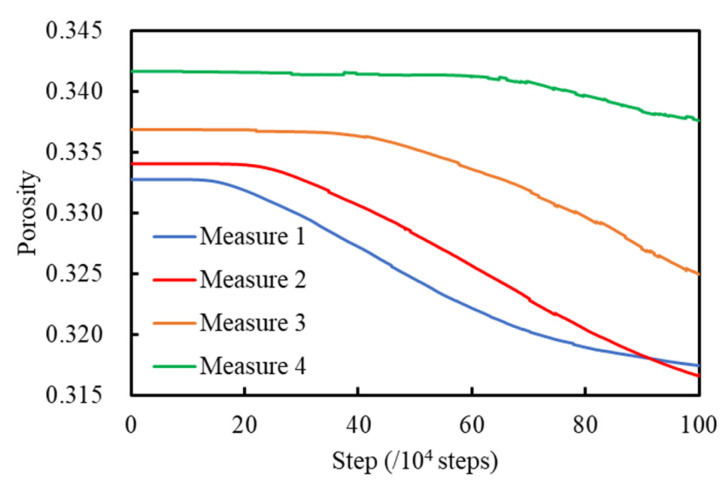
Curve of measured porosities.

When the calculation was completed, the change of porosities was recorded by 4 measure circles, as shown in Figure 22. With the support of BFR gabions, the broken bonds were distributed evenly. The soil particles in the slope gradually became denser, and the porosities measured became smaller gradually.

## 5. Discussion

**Figure 23:**
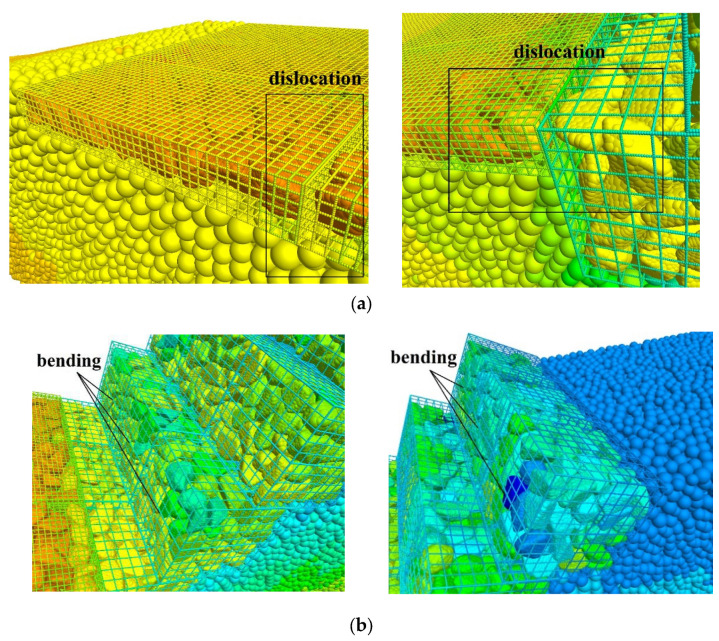
Failure of the gabion slope. (**a**) Dislocation; (**b**) Bending.

**Figure 24:**
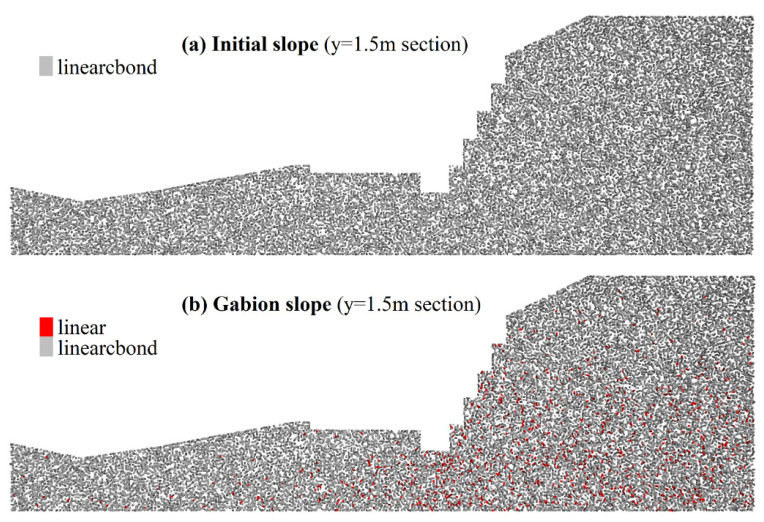
Distribution of the contact bond model. Section of y = 1.5 m.

The failure of the BFR gabion slope is mainly about the dislocation of the gabions and the bending of BFRs as shown in Figure 23. Figure 24 shows the distribution of the contact bond model with a section of y = 1.5 m. The slope does not form a sliding surface under the support of the BFR gabions. There are mainly three reasons: (1) the density of the rock blocks filled in the gabion is 1.79 times that of the soil. It is equivalent to adding cover weight on the slope surface, which is beneficial to the stability of the slope. (2) After using BFR gabions, there is friction between the rock blocks, rock blocks, cages, gabions, and soil. (3) The diameter of BFR is 4 mm to 6 mm, with better wrapping and restraining ability than the traditional wire.

In this study, the number of BFR tensile specimens is limited, and the mechanical properties of the BFR cage can be further studied. The contact problem of gabion slope protection is very complex, which can be well-simulated by PFC3D. In order to obtain better simulation results, the particle size of the soil should be smaller than that of block rock, which makes the calculation scale very large and time consuming. This paper’s refined 3D DEM model has about two million contacts and takes about 100 h. Further study is needed to adequately reduce the calculation scale to obtain the ideal simulation result. In addition, the DEM can reasonably simulate the destruction of reinforcement, and it is suitable to simulate the impact of the gabion cage damage. The current simulation does not consider cage modeling, leading to the difference from the actual situation. The gabion cage has the reinforcement of the constraint to block rocks. As a result, we cannot oversimplify the simulation. The calibration process of soil and gabion’s micro-parameters is universal, which can provide a reference for other PFC3D simulations. In this work, the BFR gabion is mainly simulated by axial load, not considering the particle breakage phenomenon of stone. However, we cannot ignore the particle breakage phenomenon in the stone cage under the impact load. We did not consider the effect of porosity in the simplified seepage simulation, but the simplified seepage simulation method proposed in this paper has certain universality. Only the forces in the x and y directions are applied for the two-dimensional model. In addition, we can apply the seepage force corresponding to a different time to simplify the simulation for transient seepage.

## 6. Conclusions

This work carries out safety risk analysis of a new structure combining BFR and the traditional gabion structure. A tensile test determined the tensile strength of BFR, and the micro-parameters of gabion and soil were calibrated. Then a simplified simulation method of seepage in DEM and the monitoring data are investigated and used to analyze the 3D BFR gabion slope. The conclusions of this study are drawn as follows:

1. The use of BFR in the gabion structure combines both advantages of BFR and traditional gabion structure. This new type of gabion structure is worthy of further promotion and application.
2. The tensile test shows that the tensile strength of the BFR is 68.22 MPa. Under the action of uniaxial compression, the BFR gabion undergoes brittle fracture. Its peak stress is 6.83 MPa, and the corresponding axial strain is 0.65%.
3. The FEM is used to calculate the hydraulic gradient to apply the seepage force for simulating the seepage effect in 3D DEM through the particle force analysis. The whole calculation process is simple and easy to program.
4. After using BFR gabions, the safety factor of the slope has increased by 25.68% compared with the original slope. The failure of the BFR gabion slope is manifested as the dislocation of the gabions and bending of BFRs.

## Figures and Tables

**Table 1:** Tensile strength of single BFR.

Sample	Tensile Force (N)	Tensile Strength (MPa)	Breaking Elongation (%)	Results
1	2567	58	-	The clamping end was broken and skidded
2	5037	114	5.8	The same with sample 1
3	7924	179	-	The same with sample 1
4	3810	86	-	Fracture of the head

**Table 2:** Basic mechanical parameters.

Material	Density (kg/m^3^)	Friction Angle (°)	Cohesion (kPa)	Elastic Modulus (Pa)	Poisson’s Ratio	Friction Coefficient with Cage
Soil	1480	14	15	5 × 10^6^	0.30	0.55
Rock block	2650	60	0	56 × 10^9^	0.20	-
Cage	2060	-	-	50 × 10^9^	0.22	0.60

**Table 3:** Micro-parameters of BFR.

Density (kg/m^3^)	Diameter (m)	Emod (Pa)	Kratio	Fric	pb_ten (Pa)	pb_coh (Pa)	emod * (Pa)	Kratio *
2060	0.005	50 × 10^9^	2.0	0.2	7.9 × 10^8^	9.0 × 10^8^	50 × 10^9^	2.0

Note: * means the parallel-bond group.

**Table 4:** Micro-parameters of soil.

Diameter (m)	Diameter (m)	Emod (Pa)	Kratio	Fric	cb_tenf (N)	cb_shearf (N)
0.10–0.14	0.35	5 × 10^6^	2.0	0.06	100.0	100.0

**Table 5:** Micro-parameters of BFR gabion.

Contact	Density (kg/m^3^)	Diameter (m)	Emod (Pa)	Kratio	Fric
A1	2650	0.08–0.10	56 × 10^9^	2.4	0.65
A2	-	-	2.8 × 10^7^	2.0	0.35

Note: A1 is the contact between rock blocks; A2 is the contact between rock blocks and BFRs.

**Table 6:** Micro-parameters of different interfaces.

Contact	Emod (Pa)	Kratio	Fric
C1	1.0 × 10^9^	1.0	0.2
C2	50 × 10^9^	2.0	0.3
C3	56 × 10^9^	2.0	0.3

## Data Availability

The data used to support the findings of this study are available from the corresponding author or the first author upon request.
